# THE IMPACT OF CONTRACT NURSE UTILIZATION ON NURSING HOME FINANCIAL PERFORMANCE

**DOI:** 10.1093/geroni/igad104.0747

**Published:** 2023-12-21

**Authors:** Akbar Ghiasi, Gani Davlyatov, Rohit Pradhan, Justin Lord

**Affiliations:** University of the Incarnate Word, San Antonio, Texas, United States; University of Oklahoma Health Sciences Center, Oklahoma City, Oklahoma, United States; Texas State University, San Marcos, Texas, United States; Louisiana State University at Shreveport, Shreveport, Louisiana, United States

## Abstract

Ensuring the financial viability of NHs is an important policy and managerial concern. The financial sustainability of nursing homes has been threatened in the recent past by high staff turnover, growing competition, low Medicaid reimbursement, and declining occupancy rates. The literature suggests that nurse staffing may have a significant impact on NH financial performance. We examined whether higher ratios of contract nurse use were associated with NHs operating margin. Employing a pooled cross-sectional observational study design, we extracted secondary data from the PBJ nurse staffing, Nursing Home Compare, Healthcare Cost Report Information System (HCRIS) Dataset, RUCA codes, and the ACS for the period 2017-2021. To test the relationship between the ratios of contract nurses and operating margin, we employed multivariate mixed-effects maximum likelihood regression. We ran three separate regressions for RN, LPN, and CNA. Our findings showed that a 10% increase in the contract nurse ratio among RNs, LPNs, and CNAs is associated with a 0.45%, 0.48%, and 0.52% decrease in operating margin, respectively. Our results demonstrate a consistent negative association between the ratio of contract nurses and nursing home operating margin. Our results are consistent with the extant literature with contract nurse staffing said to be significantly more expensive. Considering NHs operate in a financially precarious environment, the increased utilization of contract nurse staffing may pose a further threat to their financial viability, with negative implications for quality and access.

